# Emergency Medications and Equipment Indispensable for Dental Offices and Clinics in Iran

**DOI:** 10.30476/dentjods.2024.103415.2450

**Published:** 2024-12-01

**Authors:** Saeed Nemati, Shahram Hamedani

**Affiliations:** 1 Shiraz University of Medical Sciences, Shiraz, Iran; 2 Oral and Dental Disease Research Center, School of Dentistry, Shiraz University of Medical Sciences, Shiraz, Iran

**Keywords:** Emergency drug dosage, Emergency drugs, Emergency kit, Emergency management, Emergency dentistry

## Abstract

Medical emergencies, though rare, may occur unpredictably in a dental office during or after dental procedures. It may occur accidentally or subsequent to systemic problems of patients. These series of clinical events entail instantaneous management to evade any potential consequences. Basic life support measures require sufficient knowledge, expertise, skills, and equipment. Several medications and equipment are necessary to be available for immediate reach in any dental office. Health authorities of different countries usually propose these lists to dental practitioners. However, the best emergency kit is the one, which is arranged and maintained by the dentists based on their needs and easily accessible for immediate use. Considering the importance of this subject and the results yielded from the regular visits of the Dental Supervision Department (Vice Chancellor for treatment of Shiraz University of Medical Sciences) to the dental centers of Shiraz in the spring and summer of 2023, we decided to recall the importance of emergency medicines and equipment through a brief communication.

## Introduction

All members involved in a dental team have a responsibility to ensure that they are competent in delivering an effective and safe service to their patients. A reasonable performance in a medical emergency, though rare in a dental office, has a broad spectrum insinuation in including necessary equipment, relevant education, standards of care, clinical governance, risk management, and suitable clinical audit [ 1 - 2 ].

Every day, a large number of people in society, with a wide range of ages, from children to elderly patients, undergo dental treatments. Some of these people have various systemic diseases. It has been reported that about 37% of people who are referred to a dentist in the Netherlands have medical problems, the most important of which are heart-respiratory diseases, brain diseases, or a history of convulsions [ 3 - 4 ].

In terms of dental treatment measures, the prescription of local anesthetics and the pressure and stress caused by performing dental services can cause clinical symptoms and various emergencies in the patients or aggravate their systemic diseases [ 5 - 6 ]. Therefore, the dentist must have sufficient knowledge of the pathophysiology of many diseases, the effects of various drugs, and the complex structure and functions of various body systems. In this case, in addition to the diagnosis and providing an appropriate treatment plan, dentists should have sufficient competency and full preparedness for emergency cases and other related risks [ 7 - 8 ]. Moreover, nowadays, due to increased life expectancy and growing elderly populations as well as advances in dentistry, more patients with systemic problems demand their dental treatments to be performed in fewer but longer sessions. This has led to a higher number of different medicines prescribed in the dental profession, and as a result, the number of emergency cases in dental offices is increasing [ 9 ]. Emergencies are not common events in a dental office, but if they happen, they are one of the most unfortunate events that can occur in a dental office and might expose the patient and the dentist to a very dangerous situation [ 5 , 7 , 9 ]. 

The most common cases are hyperventilation, increased blood pressure, thyroid storm, asthmatic attacks, epilepsy, hypoglycemic shock, cardiac arrest, stroke, and myocardial infarction [ 1 , 5 , 9 ]. On the other hand, when dentists are asked about the actions they take in case of an emergency, unfortunately, most of them do not have a clear answer due to their lack of knowledge about emergency medicines and their use. In most instances, they do not think there is any other way but to call for an emergency (in Iran, the telephone number is 115, in USA 911). However, regardless of the compassionate efforts of colleagues in emergency settings, due to various reasons, the golden time to save the patient may have passed by the time they arrive, so the importance of familiarity with emergency medicine and related equipment for those in dental practice becomes more and more apparent. In a 10-year report in England, 20 deaths were reported due to these unfortunate events [ 4 , 10 ]. 

A dentist, as a member of the medical community, is obliged to have sufficient preparation in his practice to save the lives of patients who need medical intervention during work or even in the waiting room. On average, nearly 2% of the events that happened in the dental office were cardio-pulmonary resuscitation [ 10 ]. Other studies have shown that 15% of Australian dentists [ 11 ] and 5% of Ohio dentists have performed cardiopulmonary resuscitation on at least one patient during their career [ 12 ], and 3% of Brazilian dentists have reported cardiac arrest in their dental offices [ 4 , 13 ]. Dym [ 14 ] reported that a dental office, among the main emergency equipment, should have a variety of Ambo Bag syringes, a portable oxygen system, preferably an EKG/defibrillator device, a sphygmomanometer (child and adult sizes), as well as emergency medicines including atropine, aspirin, nitroglycerin, epinephrine, and salbutamol [ 3 , 14 ].

In a study conducted in Tabriz city in 2001, postural hypotension and vasopressor syncope were the most common emergency incidents in dental offices, and based on this research, the most needed emergency drugs were epinephrine and oxygen, and the most required supplies in the emergency kit included suction, head suction, and serum [ 3 , 15 ]. 

In our country, many other emergency cases have also been recorded in dental offices. During research conducted in Zahedan city in Iran (2014), it was found that more than half of the dentists had faced at least one medical emergency in their past year of practice [ 3 , 16 ]. 

Likewise, many studies have been performed in different provinces and cities of Iran, concerning dentists’ awareness of emergency equipment and medicines in dental offices and clinics [ 17 - 19 ]. 

These studies have mainly one major conclusion that there is a clear need for educating dentists to increase their preparedness for emergency management [ 20 ].

A cross-sectional study performed in Shiraz reported that the dentists' knowledge about emergencies was average, while the knowledge was higher in those who had taken part in emergency workshops [ 20 ].

Considering the importance of the above-mentioned matter and the results yielded from the regular visits of our control team (Dental Supervision Department, Vice Chancellor for Treatment of Shiraz University of Medical Sciences) to the dental centers of Shiraz in the spring and summer of 2023, we decided to recall the importance of emergency medicines and equipment through a brief communication. In this short communication, some emergency medicines and equipment that are required in dental offices and dental clinics are presented and subsequently discussed (Table 1).

**Table 1:** Critical medications in the dental emergency and their dosages [ 1 , 6 , 8 - 9 , 21 - 23]

		Drug classification	Indications and usage	Dosage forms	Dosage and administration
**Epinephrine**	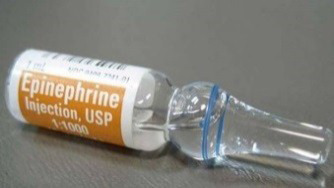	Adrenergic, bronchodilator, heart stimulant, vasopressor, auxiliary drug in local anesthesia	Epinephrine injection is a non-selective alpha and beta-adrenergic receptor agonist, indicated in the emergency treatment of allergic reactions (Type I) including anaphylaxis, as well as asthma unresponsive to salbutamol and cardiac arrest	INJ: 1 mg/1ml (1:1000 solution) injection, for IM or subcutaneous (SC) use and 1: 10,000 dilutions for IV	In anaphylaxis: Adults (0.1 mg IV., or 0.3–0.5 mg IM) and Pediatrics (1:1000 0.05-0.3 mg IM or1:10,000 0.01 mg/kg IV), In asthmatic attack unresponsive to salbutamol: Adults (0.1 mg IV, or 0.3–0.5 mg IM.), In cardiac arrest: adults (1 mg IV)
**Hydrocortisone**	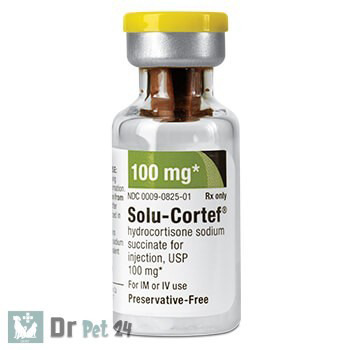	Corticosteroid, steroidal anti-inflammatory, to replace adrenocortical	Reduction of inflammation, shock, symptomatic treatment of allergic disorders, and allergic shock	INJ: 100, 200 mg	Adults: 100 mg at first, then 250 to 500 mg IM every 6-2 h in acute cases, Children: 1 mg/kg IM or IV Direct intravenous injection or diluted with normal saline or 5% dextrose.
**Atropine**	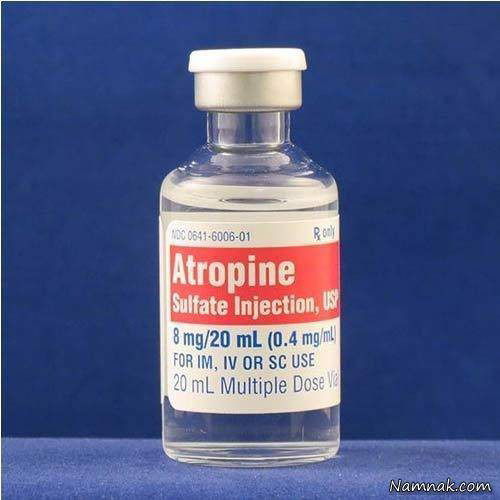	Anticholinergic, antispasmodic, antiarrhythmic	Clinically significant bradycardia with a heart rate of less than 60 beats per minute	INJ: 0.5 mg/1 ml; 10 mg/10 ml; 20 mg/2 ml; 2 mg//8 ml	Adults: 0.5 to 1 mg IV and every 3-5 min if needed until the heart rate reaches 60, repeat until it reaches a maximum of 3 mg. An injection of less than 0.5 mg causes vagal stimulation in the brain and decreases heart rate. In children (0.02 mg/kg IV or 0.04 mz/kg (IM/SC)
**Diazepam**	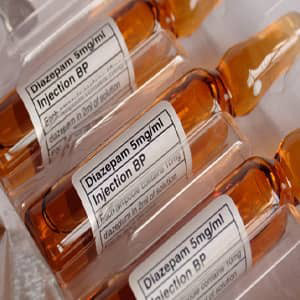	Benzodiazepine, sedative, anticonvulsant, hypnotic, muscle relaxant, sedative (anesthesia)	Status epilepticus, seizures, anxiety, stress, alcohol deprivation syndrome	Tablets: 2,5,10 mg; Suppositories: 5, 10 mg; Syrup: 2 mg/5 ml; Rectal tube: 5, 10 mg; INJ: 10 mg/2 ml	In Adults: (2-10 mg) orally 2-4 times per day or 5-10 mg IM or IV. If necessary, this dose should be repeated 3-4 hours later. Rectal (0.2 mg/kg), In pediatrics: rectal (0.3-0.5 mg/kg) IM (0.1-0.2 mg/kg)
**Dexamethasone**	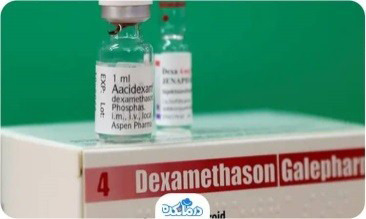	Corticosteroid	Treatment of inflammatory and allergic conditions, autoimmune diseases such as lupus erythematosus, allergy, pemphigus, asthma, urticaria, treatment of joint rheumatism, anaphylactic shock, Inflammation of eyes, skin, joints	INJ: 8 mg/2 ml (as disodium salt); Tablets: 0.5 mg; Elixir: 0.5 mg/5 ml	For adrenal crisis anaphylaxis: In adults: 4 mg (IM), In pediatrics: 0.25-0.5 mg/kg (IM)
**Lidocaine**	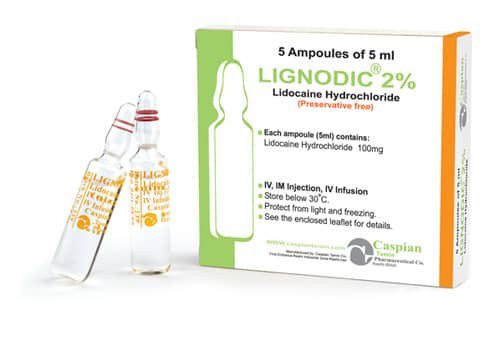	Antiarrhythmic, local anesthesia	Premature ventricular tachycardia, cardiac arrhythmia	INJ: 2%	Adults: 50 or 100 mg (IV) or 1-1.5 mg/kg (IV/IO/ET) at a rate of 20-25 mg per minute
					Pediatrics: Up to 100 mg IV) or 0.5-1 mg/kg (IV/IO/ET), Note: The dose used in children might be different, referring to pharmaceutical books is recommended
**Naloxone**	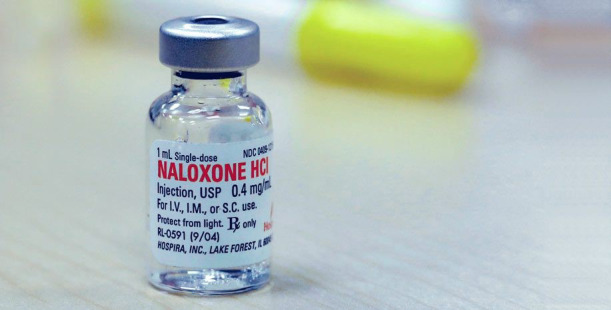	Narcotic antagonist	To relieve respiratory depression caused by opium-like drugs, to treat opioid poisoning and opioid overdose	INJ: 0.4 mg/ml	Adults: 0.4 mg (IV) Dilute an ampoule with 4 ml of distilled water or normal saline and inject less than half a cc every 2 min until the respiratory symptoms are relieved.
					Pediatrics: 0.1 mg/ml (IV) or 0.01 mg/kg (IM)
**Dextrose**	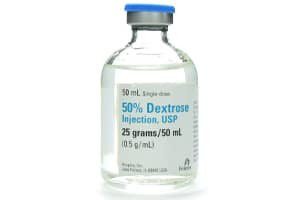	Fluid and nutrient replenisher	Hypoglycemia, all hypoglycemia caused by cold, lack of energy caused by dehydration, anorexia and ketosis, prolonged hunger	Dextrose 50% hypertonic vial, IV infusion solution	In adults: 50% dextrose (100 ml) or 1 mg/kg of body weight, if there is no response to the treatment, it should be repeated after 12 h. In children: 25% dextrose 1-4 ml/kg
**Salbutamol spray**	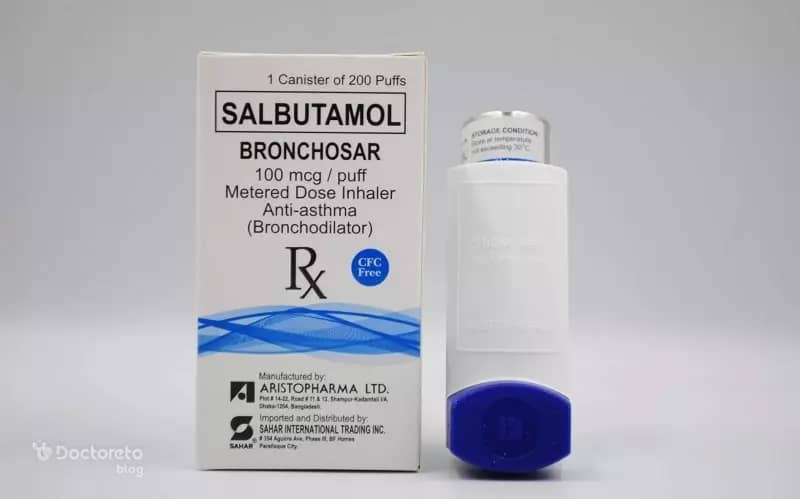	Sympathetic mimic drugs, bronchodilator	Main and fast-acting medicine in asthma attacks and maintenance medicine in chronic asthma	Metered dose inhaler	Adults: (100 g per actuation, 2-3 inhalations/1-2 min), 1-2 puff of spray and repeat it every 4-6 h. First, shake the spray well, put the spray nozzle in the mouth, and let the lips close tightly around it so that the air does not enter your mouth in another way. Take a deep breath and use the spray at the same time. It is important to press the spray just once at the moment you start breathing so that the medicine penetrates deep into the lungs along with inhaled air. After 11 sec, slowly exhale and start normal breathing. Wait at least one minute if you need to use it again.
					Pediatrics: 100 μg per actuation, 1 inhalation/1-2 min
Nitroglycerin sublingual tablets and spray	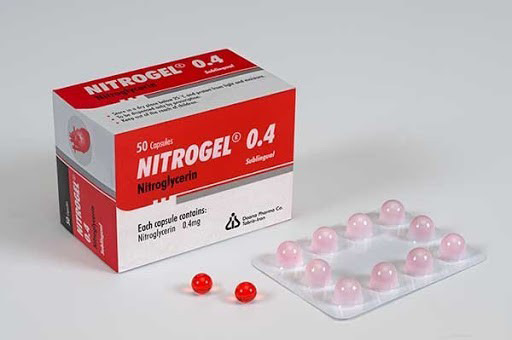	Anti-anginal, vasodilator, anti-hypertensive	Hypertension control, chronic angina attacks, angina pectoris, and reduction of acute chest pain	Sublingual tablets (pearls), 0.3, 0.4, 0.6 mg; Spray	In Adults: (0.4 mg q 5 min 2-3 times) A capsule or pearl should be taken under the patient's tongue immediately after starting the pain. No more than 3 total tablets should be taken within 15 min. Besides, the patient can use this product 5-10 min before exposure to stress or activity that causes pain in the chest. For sprays, one or two spray puffs (400 μg per actuation) can be used instead of each dose of medicine, Pediatrics: Amyl nitrate 0.3 ml Vaporole
	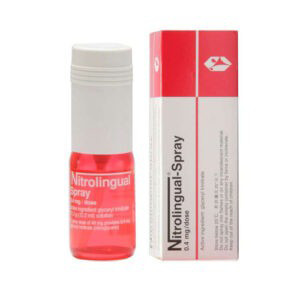				
**Aspirin (ASA)**	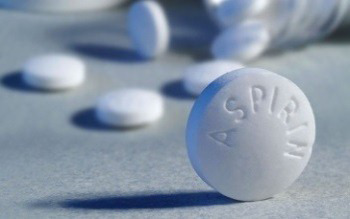	Salicylate, non-narcotic pain reliever, non-steroidal anti-inflammatory, antipyretic, platelet aggregation inhibitor	thromboembolic disorders, transient ischemic attacks, to reduce risk of heart attack, a history of myocardial infarction (MI) or heart attack and unstable angina	Tablets: 80 mg (chewable); 100, 325, 500 mg	Adults: in ischemic heart attacks, it should be used quickly by the patient with doses of 80 to 325 mg
					Pediatrics dose: 5-10 mg/kg q 6 h
**Metoclopramide**	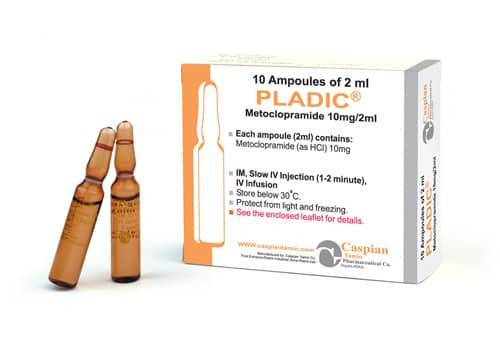	Gastrointestinal stimulant, antiemetic, cholinergic	Antiemetic	INJ: 10 mg/2 ml; Tab: 10 mg	The injection route is IM, the tab (10 mg) should be consumed 30 min before the meal
**Chlorpheniramine**	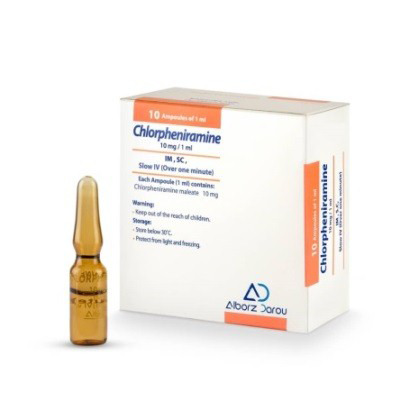	Antihistamine, antiallergic, sedative	Rhinitis )most common), mild allergy, common colds, urticaria, and angioedema	Ampule, tablet, drop, syrup, ointment	Adults: 10-20 mg (oral/IM)
					Children: 1-2 mg q 6 h max 8 mg/day
**Diphenhydramine**	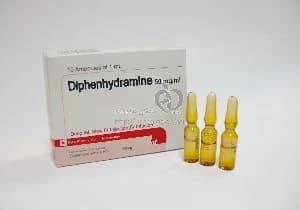	Antihistaminic, motion sickness, anti-parkinsonism	Allergic reactions to blood or plasma, in anaphylaxis (as an adjunct to epinephrine and other standard measures after controlling the acute symptoms), uncomplicated allergic conditions of the immediate type when oral therapy is impossible or contraindicated	IV or IM only (When the oral form is impractical) Dosage should be individualized	Adults: 10 to 50 mg IV at a rate generally not exceeding 25 mg/min, or deep IM; 100 mg if required; maximum daily dosage is 400 mg.
					Pediatric other than premature infants and neonates: 5 mg/kg/24 h or 150 mg/min /24 h Maximum daily dosage is 300 mg. Divide into four doses, administered IV at a rate generally not exceeding 25 mg/min, or deep IM

IV: intravenous; IM: intramuscular; SC: subcutaneous; INJ: injectable dosage form. ; ET: direct endotracheal tube administration

Please note: These are just example products, and the same medicines manufactured by other companies could be equivalently used.

It should be noted that the medications that must be available in the emergency box in the dental office are not many, but in large and well-equipped centers and dental clinics in which more complex surgeries and treatments are performed and have a high volume of patients, the simple emergency kit and the box is not enough and an emergency trolley is requisite [ 2 , 8 ].

Likewise, in the offices where extensive surgeries are performed, it is preferable to have an emergency trolley. In these centers, a trained and experienced nurse must be present to use the emergency trolley items during all the hours when the center is operating; they should also know how to store them properly [ 5 ]. 

The emergency trolley is a mobile unit designed for the essential purpose of cardiopulmonary resuscitation and other emergency cases. This trolley should have already been equipped with all the medicines and necessities needed by the emergency team. This mobile unit should be placed in the strategic places of the medical center or emergency room, well accessible for the trained medical team, so that could be quickly brought to the patient's bed during cardiopulmonary arrest and other emergencies. Dentists are advised to prepare a special kit or purchase a primed kit that contains emergency medications so that they are well-trained in how to use them with the quantity that has been recommended by the regulatory bodies in their own country. 

### What emergency medicines should be available in a dental office?

The required medicines are tabulated with their relevant pictures in Table 1; however, a brief discussion is presented as follows [ 8 , 21 - 22 ]. 

### Epinephrine

Epinephrine is undoubtedly the most important drug in different pharmaceutical forms that is used in dentistry for local anesthetics; however, in the emergency kit and box, it should be in a pharmaceutical form other than the usual dental carpule. This medicine, which is stored as 1 in 1000 in syringes ready for injection, is a sympathetic stimulant drug that plays a vital role in saving the patient's life in anaphylactic shock, which is the most dangerous emergency in a dental office. This medicine is also used in dangerous asthma attacks. It can be used by injection, intramuscularly, and subcutaneously. In cardiac resuscitation, epinephrine is injected intravenously or directly into the heart, which is not part of the emergency treatment of the dental office and requires a professional emergency resuscitation team. The shelf life of this medicine is limited and attention should be paid to renewing the medicine ready for injection at a certain time.

### Hydrocortisone

A corticosteroid such as hydrocortisone may be prescribed for the prevention of recurrent anaphylaxis. Hydrocortisone would also help in the management of an adrenal crisis. However, their relatively slow onset of action (even one hour when administered IV) is considered as their limitation. The prototype for this group is hydrocortisone, which may be administered in a dose of 100 mg as part of the management of these emergencies [ 21 ]. Patients with primary adrenal insufficiency should be advised to bring their emergency hydrocortisone injection kit to all dental appointments, as well as their personalized adrenal crisis letter which provides medical treatment guidance. The recommended dose, which should be presented in the patient’s letter, depends on the patient’s age; Adults: 100 mg, Children six years of age or older: 50–100 mg, Children one to five years of age: 50 mg; Infants up to one year of age: 25mg [ 1 ]. 

### Dexamethasone

Dexamethasone is used as an antiemetic and for the treatment of severe allergies, pruritus, asthma, bronchospasm, and postoperative edema. With a slow onset of action, its classical dose is 4 to 12 mg IV. Care should be taken for patients with pre-existing infections, peptic ulcers, or hyperglycemia [ 23 ]. 

### Atropine

Atropine is an anticholinergic medicine that can be available in the emergency medicine kit as an injection. In cases of bradycardia and cardiac arrest caused by intense vagal stimulation, asystole, pulseless electrical activity with a heart rate less than 60 is very effective, and in higher doses, it can be used as an antidote for organophosphorus poisoning.

### Benzodiazepines (Diazepam)

Benzodiazepines are administered to manage prolonged or recurrent seizures (status epilepticus). IV diazepam 5-10 mg is fast in impeding all types of seizures. An alternative treatment for seizures is midazolam or lorazepam IM/IV. Patients should be monitored vigilantly after administration to check for any respiratory depression and sedation. Recently, buccal midazolam is also suggested to treat seizures [ 6 ]. 

### Lidocaine 1 and 2%

Lately, lidocaine has come back into focus for the treatment of acute sustained ventricular tachyarrhythmias [ 24 ]. 

### Naloxone

Naloxone is a specific opioid antidote that reverses the opioid-induced respiratory depression. This medicine should be used for the emergency management of opioid (morphine) overdose. As a narcotic antagonist; it is employed to relieve respiratory depression caused by opium-like medicines to treat opioid poisoning [ 6 ] after administration of naloxone, patients should be monitored in the office for 1 hour to rule out re-sedation. A typical dose is 0.1-mg increments [ 23 ]. 

### Bronchodilators (salbutamol Spray, aminophylline)

Salbutamol is a short-acting selective beta-2 adrenergic receptor agonist. This is the preference for bronchospasm with acute asthmatic episodes [ 6 ]. It is suggested 1-2 puffs of spray for adults every 4-6 hours. The procedure for usage is as follows. First, shake the spray well, put the spray nozzle in the mouth, and let the lips close tightly around it so that the air does not enter your mouth in another way. Take a deep breath and use the spray at the same time. It is important to press the spray just once at the moment you start breathing so that the medicine penetrates deep into the lungs along with inhaled air. After 11 seconds, slowly exhale and start normal breathing. Wait at least one minute if you need to use it again. After use, put the mouth cover back on to prevent dust from entering, 2 to 6 puffs can be used in asthma attacks.

Aminophylline, whose injectable form is 250mg ampoule in 10 ml, is a suitable medicine to be present as another bronchodilator in the emergency kit. In acute bronchospasm (following an allergic reaction) and asthma attacks, it can be life-saving [ 5 ]. 

### Nitroglycerin sublingual tablets and spray

Nitroglycerin is the drug of choice to treat acute angina or myocardial infarction. It has a fast onset of action. It is presented as oral and transmucosal preparations (spray), transcutaneous patches, and IV solutions. Sublingual tablets or sprays are appropriate forms for dental settings. Sublingual tablets should be freshly opened because of their short shelf life.

These tablets should not be swallowed, crushed, or chewed, but in cases of hypertension or angina pectoris, one pearl capsule of nitroglycerin should be administered under the patient's tongue immediately after starting the pain. For patients with xerostomia, a small sip of water before placing the tablet under the tongue may aid dissolution of the tablet.These tablets can be used three times within 15 minutes. In addition, the patient can use this product 5-10 minutes before exposure to stress or activity that causes pain in the chest. In addition, one or two spray puffs can be used instead of each dose of medicine [ 1 , 6 , 22 ]. 

### Aspirin (ASA)

Aspirin reduces overall mortality from acute myocardial infarction by preventing further clot formation. It is employed in thromboembolic disorders and transient ischemic attacks and is used to reduce the risk of heart attack. It is usually on the medication list of patients with a history of myocardial infarction or heart attack and unstable angina. Aspirin is a platelet aggregation inhibitor and can prevent further damage by diluting the blood clot. It should be noted that aspirin should not be used after dental surgery. On the other hand, dentists should pay attention to patients who have a history of daily aspirin use. If they decide to perform dental services with bleeding surgeries and tooth extraction, they must stop taking aspirin 3-5 days before performing the service, after consulting with the related specialist. Co-administration of nitroglycerin with high-dose aspirin (1000 mg) will lead to amplified exposure to nitroglycerin. The vasodilation and hemodynamic effects of nitroglycerin may be increased by simultaneous administration of nitroglycerin with high-dose aspirin [ 1 , 5 , 8 ]. 

### Metoclopramide

Metoclopramide is used to avert nausea and vomiting after procedural sedation. The recommended dose is 10 mg. It can cause movement disorders. It should not be administered in patients with Parkinson's disease. It can also prompt neuroleptic malignant syndrome, which shows up as high fever, confusion, rigid muscles, and autonomic imbalance. To manage this situation, impeding the agent, rapid cooling, dantrolene, and benzodiazepines is suggested [ 23 ]. 

### Injectable antihistamines (diphenhydramine, chlorpheniramine)

An antihistamine is administered for the management of allergic reactions. Oral administration of antihistamines is prudent for mild non-life-threatening allergic reactions whereas parenteral administration is requisite for life-threatening reactions. An injectable histamine blocker is necessary for a dental emergency kit. Two injectable agents may be considered, either diphenhydramine or chlorpheniramine. They may be administered as part of the management of anaphylaxis or as the sole management of less severe allergic reactions, particularly those with primarily dermatologic signs and symptoms such as urticaria.

Diphenhydramine is the best choice for a dental emergency kit, which can also be used in milder allergic reactions (compared to anaphylactic shock). After epinephrine, it is considered an important medicine in anaphylaxis shock. In a dose of 50 mg, it is used intravenously or intramuscularly. The paediatric dose of diphenhydramine dose is 1-1.25 mg/kg every 6 hours.

The most common use of chlorpheniramine is in the disease known as rhinitis, which is chronic inflammation of the nose or inflammation of the mucous membranes of the nose. It is also used in common colds, allergies, urticaria, and angioedema. Recommended doses for adults are 10 to 20 or 25mg of chlorpheniramine [ 6 , 21 - 22 ]. 

### Glucose and oral carbohydrates

In the office, there should be access to sugar for patients' emergencies. Generally, office assistants usually know how to make sugar syrup; however, there are many problems when your patient has lost consciousness and cannot drink sugar water. In this case, dextrose or glucagon serum (1mg) is injected into the patient. Oral carbohydrates such as fruit juice or non-diet soft drinks are used to control early hypoglycemia in conscious patients. Oral carbohydrates can quickly restore blood sugar levels. A missed meal might likely be the cause of hypoglycemia in insulin-dependent diabetic patients [ 6 ]. A vial of 50% hypertonic glucose (dextrose 50%) is used to increase glucose levels in hypoglycemia (caused by cold, lack of energy caused by dehydration, anorexia, and ketosis, prolonged hunger). If patients are unable to swallow, IV access should be employed [ 23 ]. 

In adults, 1 ml/kg of body weight of 50% dextrose solution is recommended. If there is no response to the treatment, it should be repeated after 12 hours. In children, the recommended dosage is 5ml/kg of body weight of 20% dextrose solution. 

### Oxygen

Oxygen is used for the treatment of hypoxemia, which is frequent in several medical emergencies. A supplemental oxygen delivery system (oxygen capsule) and its complete accessories are needed and indicated for all dental emergencies. Oxygen can be used in almost all dental emergencies and it does not pose any danger to the patient. It is very beneficial for dentists to help patients in case of shortness of breath during work (especially in the post-COVID-19 situation). A portable full-sized cylinder should be available for the patient's oxygenation until the arrival of emergency services. Oxygen is delivered with a clear full-face mask with a flow rate of 10 l/min for the impulsively breathing adult patient and 3-5 l/min for breathing children. Bag-valve-mask device is necessary for providing oxygen for the unconscious and apnoeic patient at a flow rate of 10-15 l/min, and in case of positive pressure device usage, the flow rate should not go beyond 35 l/min for adults [ 6 ]. The oral surgery office must also be equipped with a bag valve mask with a full face mask to permit positive pressure ventilation [ 23 ]. 

### Other Necessary Medications

Verapamil, which is used in the treatment of tachycardia, anti-angina, and anti-hypertension, morphine, which is used to control the pain of myocardial infarction and acute pulmonary edema, dopamine (in severe hypotension), amiodarone (the most effective anti-arrhythmic medicine, dobutamine (increasing cardiac output), heparin (after MI, thrombosis and embolism), magnesium sulfate (pregnancy hypertension and acceleration of bowel movements), propranolol (antiarrhythmic and antianginal), furosemide and hydralazine (diuretic and hypertensive), phenytoin (anticonvulsant and antiarrhythmic), nitroprusside (blood pressure control in anesthesia) are other emergency medicines, which, of course, require the presence of a nurse or an experienced doctor in the field of emergency for usage.

Tools and equipment needed in emergencies in dental offices and centers [ 1 , 9 - 10 , 21 - 22 ].

In addition to the emergency kit, sometimes in dental offices, special equipment is needed, and in addition to the emergency kit and medicines, dental practitioners should also prepare special equipment and supplies for dental offices. These tools and equipment are portable oxygen cylinders (E size) with regulators, supplemental oxygen delivery devices, nasal cannula, Non-Rebreathing mask with an oxygen reservoir, nasal hood, bag-valve-mask device with an oxygen reservoir, and oropharyngeal airways (adult sizes 7, 8, 9cm) [ 5 ]. 

Dentists with related training may embrace advanced airway devices in their emergency kits. Advanced training and sufficient clinical experience are needed for their indications, techniques to employ, and warranting correct placement of these devices. Endotracheal intubation is performed with the employment of a laryngoscope and an endotracheal tube. Supraglottic devices such as laryngeal mask airway are currently well-liked in airway rescue. For parenteral injection, tourniquet, and angiocath, different syringes (1, 2, and 5ml) are necessary. If possible, automatic defibrillator (AED) should also be included as dental emergency equipment, particularly in oral surgery clinics.

## Conclusion

Due to the lack of emergency equipment, kits, and medicines in many dental offices and centers, or the incompleteness or out datedness of most of the available kits and medicines, the need for full supervision by the monitoring teams is imperative. Additionally, granting medical emergency courses for dentists and providing the relevant course unit for dental students are emphasized. On the other hand, the inclusion of necessary training in general dentistry courses to increase the scientific and practical ability of future dentists in the field of medical emergencies and providing conferences and retraining programs and workshops should be considered. Moreover, preparing educational brochures, devising a list of required medicines and necessary equipment in the dental offices, and adequate supervision in their provision for working dentists should be at the top of the monitoring programs for authorities. A poster, listing the basic life support measures, is also recommended, which should be notably demonstrated in the dental office.
